# Case Report: Metastatic benign fibrous histiocytoma: a case series and review of diagnostic and therapeutic challenge

**DOI:** 10.3389/fonc.2025.1621760

**Published:** 2025-11-17

**Authors:** Xuesi Liu, Zhengwang Sun, Huajian Wu, Qingrong Ye, Chunmeng Wang, Yangbai Sun, Wangjun Yan

**Affiliations:** 1 Fudan University Shanghai Cancer Center, Department of Musculoskeletal Surgery, Shanghai, China; 2 Shanghai Medical College of Fudan University, Department of Oncology, Shanghai, China; 3 Department of Orthopedic, Fudan University Shanghai Cancer Center Xiamen Hospital, Xiamen, China

**Keywords:** benign fibrous histiocytoma, metastasis, recurrence, case report, treatment

## Abstract

**Background:**

Benign Fibrous Histiocytoma (BFH), also known as dermatofibroma, is a common benign mesenchymal tumor of the skin. Although typically non-aggressive, rare cases of metastatic BFH pose diagnostic and therapeutic challenges.

**Methods:**

This study reviews 10 cases of metastatic BFH diagnosed at Fudan University Shanghai Cancer Center between 2009 and 2024, with a detailed examination of three cases involving recurrent and metastatic behavior. Immunohistochemical markers and radiological imaging findings were analyzed to characterize tumor progression and treatment responses.

**Results:**

The cases highlighted distinct patterns of recurrence and metastasis, including lymphatic and distant organ involvement. Immunohistochemical analysis demonstrated variable expression of markers such as Vimentin, CD68, CD163, Ki67, and SMA.

**Conclusions:**

Metastatic BFH remains a rare but clinically significant condition. Early recognition, accurate histopathological diagnosis, and multimodal therapies are crucial for effective management.

## Introduction

Benign Fibrous Histiocytoma (BFH), also known as dermatofibroma, is a common mesenchymal tumor of the skin. BFH is a benign tumor arising from fibroblasts and histiocytes affecting the dermis and subcutaneous soft tissues (1). It typically presents as an isolated cutaneous nodule that is firm or cystic, skin-colored or brown, later developing into a polypoid nodule or raised plaque (2). BFH primarily affects adults and is frequently found on the extremities (3–5). There are multiple pathological subtypes of BFH, including the classic dermal FH, as well as cellular, aneurysmal, and atypical variants. Metastasis of BFH is extremely rare, with only a few reports documented in the literature (2, 5–9). This paper summarizes 10 cases of metastatic BFH diagnosed at Fudan University Shanghai Cancer Center from 2009 to 2024, with detailed reports on three cases. In this article, we use BFH as an umbrella term that includes the conventional cutaneous form and a range of histologic variants. These variants comprise cellular, aneurysmal, and atypical forms, as well as less common lipidized, palisaded, and myxoid patterns. By low-grade malignant fibrohistiocytic tumors, we refer specifically to atypical fibroxanthoma (AFX) under contemporary criteria, which is classified as BFH in this study.

## Case presentation

Among the ten cases reviewed, three were classified as cellular variants, and one as a hemangioma variant. The primary tumor sites included two cases in the head and neck, four in the limbs, one in the chest wall, one in the shoulder, one in the testis, and one with an unknown origin. Metastatic patterns included five cases with pulmonary metastases, five with lymph node metastases, two with subcutaneous metastases, and one with pleural metastasis (**Table 1**).

**Table 1:** Clinical information of ten patients with benign fibrous histiocytoma metastasis.

No	Gender	Age	Primary site	Site of metastasis
1	F	52	Unknown	Lung
2	F	48	Thigh	Lymph node
3	F	57	Arm	Lung
4	F	20	Chest wall	Lymph node
5	F	28	Jaw	Lung
6	F	28	Neck	Lung/Mutiple subcutaneous metastases
7	M	27	Arm	Lymph node
8	M	55	Shoulder	Pleura
9	M	83	Testis	Pelvis/Lung/Mediastinum/Lymph node
10	M	19	Foot	Lymph node/Pelvis

### Case 1

A 27-year-old male discovered a 0.5 cm subcutaneous nodule on his left upper arm in May 2021 (**Supplementary Figure S1**). The nodule gradually enlarged to form a 2 cm red mass protruding from the skin. In August 2021, he underwent “left shoulder mass excision,” and postoperative pathology confirmed the diagnosis of benign fibrous histiocytoma. Immunohistochemistry showed positive results for Vimentin, CD68, CD163, S100 (partial), P16 (partial), CD34, INI1, and Ki67 (5%) with negative results for SSTR2, SOX10, STAT6, EMA, CK8/18, CK19, E-cad, ALK-1A4, and ALK (D5F3).

In February 2022, a left axillary nodule was detected, and he underwent “left axillary lymph node dissection,” with pathology confirming metastasis to two left axillary lymph nodes. In April 2022, a second surgery for “extended excision of the left upper arm tumor bed” showed a cellular fibrous histiocytoma with clean resection margins. Immunohistochemistry revealed positive Vimentin, b-catenin, Bcl-2, SMA (slightly), CD163 (partial), CD68 (slightly), and Ki67 (10%) and negative results for Desmin, CD34, S100, STAT6, CK, and TLE1.

**Figure 1:**
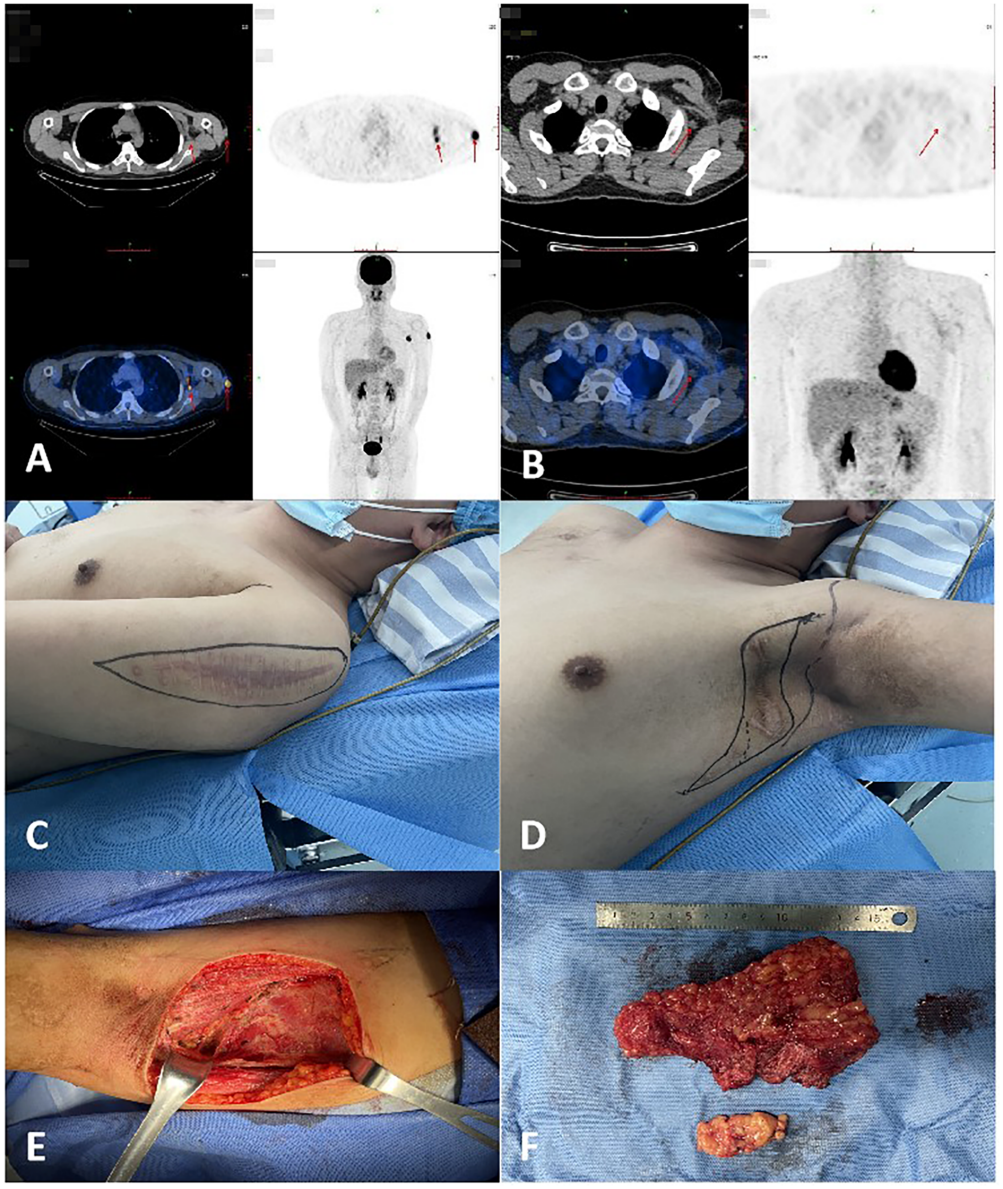
**(A)** The patient’s PET-CT results indicated lymph node metastasis in the left axilla in January 2023. **(B)** The patient’s PET-CT results revealed left axillary lymph node metastases in April 2023. **(C)** Preoperative photograph of the recurrent tumor mass in the patient’s left upper arm awaiting excision. **(D)** Preoperative photograph of the metastatic lymph nodes in the patient’s left axilla awaiting dissection. **(E)** Intraoperative photos of the patient. **(F)** Photos of the removed mass.

In January 2023, the tumor recurred (**Figure 1A**). In February 2023, further excision of the left upper arm and axillary tumor was performed, confirming lymph node metastases. Immunohistochemistry showed positive Vimentin, CD68, CD163, S100 (scattered), and Ki67 (5-30%) and negative CD34, SMA, CD117, DOG1, and STAT6.

In April 2023, MRI and PET-CT scans revealed left axillary lymph node metastases (**Figure 1B**). The patient underwent five cycles of chemotherapy with doxorubicin and ifosfamide, ending in July 2023. Despite treatment, recurrence occurred in June 2023, requiring further excision in August 2023 (**Figures 1C–F**). MRI in May 2024 suggested possible local recurrence. The patient remains under regular follow-up and disease monitoring.

### Case 2

A 28-year-old female initially presented in 2012 with a mass in the right occipital region, which was surgically excised (**Supplementary Figure S2**). Pathology indicated a low-grade malignant fibrohistiocytic tumor. Additional surgeries were performed in 2014 and 2016, followed by left cervical metastasis surgery in 2017 and right cervical and submental metastasis surgeries in 2020. Pathology described the tumors as locally invasive or low-grade malignant fibrohistiocytic tumors. Immunohistochemistry results included positive results for H3K27ME3 and CD10, with negative results for S100, Desmin, CD34, and SMA.

**Figure 2:**
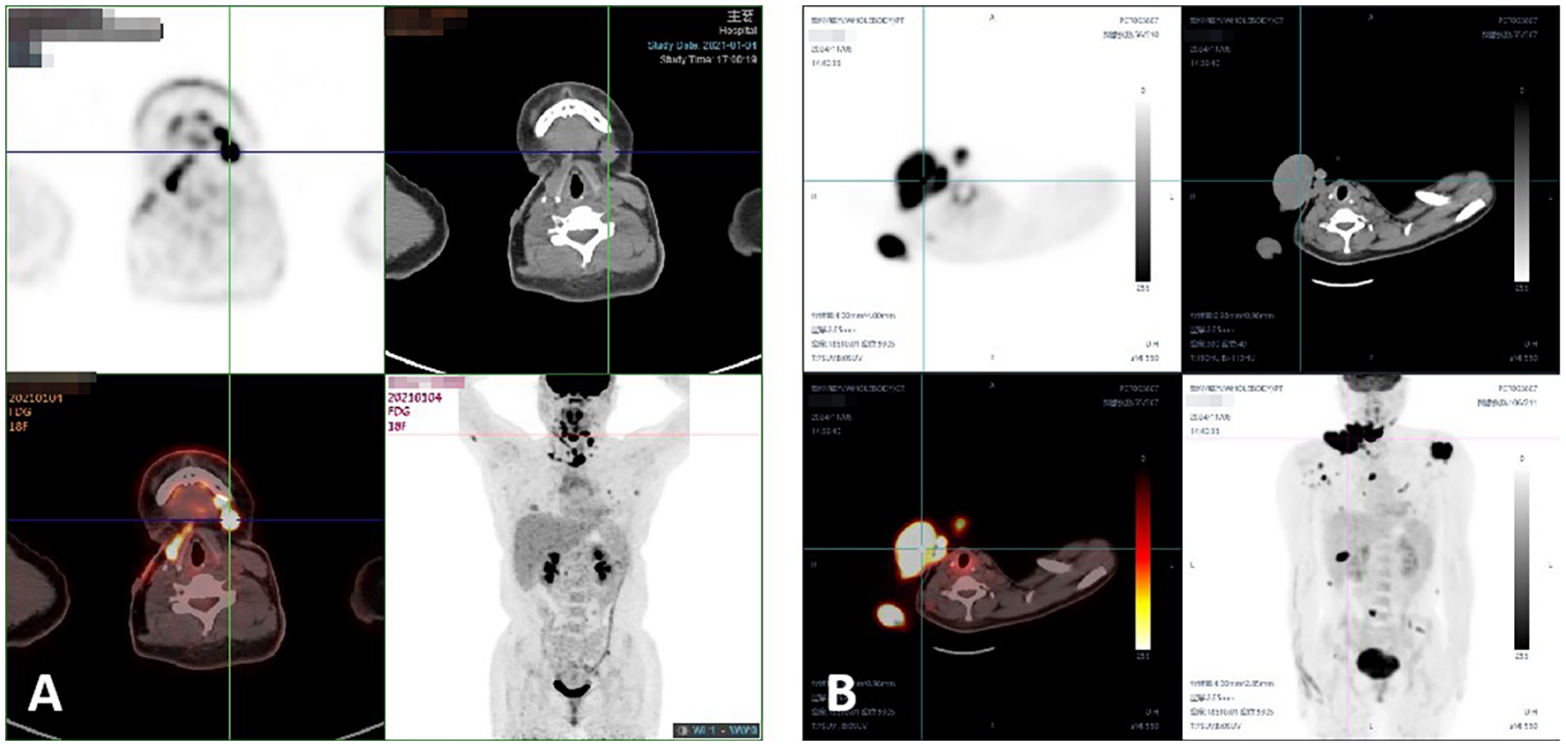
**(A)** The patient’s PET-CT results indicated multiple subcutaneous metastases in January 2021. **(B)** The patient’s PET-CT results revealed multiple subcutaneous and lung metastases in November 2024.

In November 2020, a firm 2 × 2 cm mass was noted in the submandibular region. PET-CT in January 2021 showed multiple subcutaneous nodules, predominantly in the neck and chest, with increased FDG metabolism, suggesting tumor infiltration (**Figure 2A**). The patient had weakened leg strength and difficulty walking. Surgery in January 2021 excised neck, supraclavicular, and submandibular tumors, confirmed as cellular fibrohistiocytic tumors. Immunohistochemistry results included negative SMA, CD34, Desmin, Calponin, Caldesmon, and positive Ki67 (30%).

In April 2021, radiotherapy was applied to the left clavicular region. Targeted therapy with anlotinib began in April 2024. By November 2024, recurrent and metastatic lesions were detected in multiple areas, including the neck, back, chest wall, and thighs. PET-CT revealed diffuse pulmonary nodules with mild FDG uptake, suggesting metastases (**Figure 2B**). A combination of anlotinib and PD-1 inhibitors was recommended, along with ongoing follow-up.

### Case 3

In 2020, a 19-year-old male developed a tumor in his right foot, which was surgically excised (**Supplementary Figure S3**). Recurrence in July 2022 led to a second surgery. In September 2023, a third recurrence required extensive excision, skin grafting, and reconstruction. Pathology suggested a low-grade malignant histiocytic tumor with clear margins. Immunohistochemistry showed positive results for CD1α (slightly), P16 (partial), CD34 (vascular), CD163, PGM1, and Ki67 (20% positive) with negative results for SMA, Desmin, MyoD1, ALK, S100, Clusterin, CD23, AE1/AE3, CD117, F8, and BRAF.

**Figure 3:**
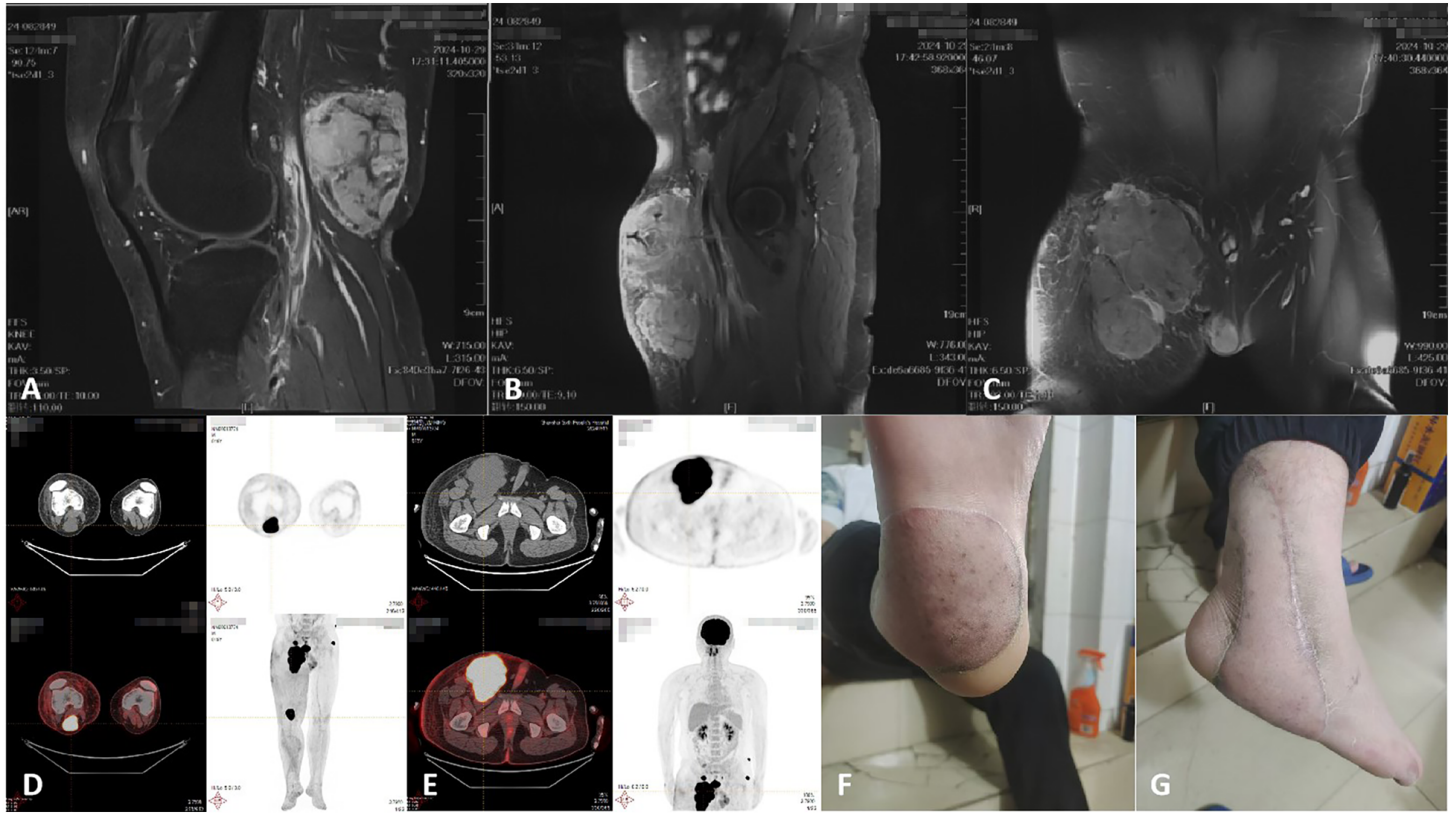
**(A-C)** MRI revealed a right groin mass in August 2024. **(D)** The patient’s PET-CT results indicated lymph nodes metastasis in the right popliteal fossa in September 2024. **(E)** The patient’s PET-CT results revealed subcutaneous metastasis in left pelvic wall in September 2024. **(F, G)** Photograph of the right foot mass at the time of the patient’s visit in November 2024.

By August 2024, the patient experienced leg swelling and a palpable right groin mass. Biopsy confirmed a metastatic low-grade malignant histiocytic tumor. Immunohistochemistry results included positive OCT2 (partial), CD163, and Ki67 (20%) with negative S100, Desmin, CD1α, Langerin, MPO, CD4, and CD45. MRI revealed a right groin mass (**Figures 3A–C**). PET-CT showed multiple enlarged lymph nodes in the right popliteal fossa, inguinal region, and iliac fossa, with the largest measuring 126 × 94 mm (**Figure 3D**). Additionally, two nodular lesions were observed under the skin of the left pelvic wall (**Figure 3E**).

Chemotherapy with doxorubicin and ifosfamide, combined with anlotinib, was administered for two cycles. In November 2024, the patient presented with a recurrent tumor in the right foot and commenced treatment with a combination of temozolomide and anlotinib (**Figures 3F–G**). Following treatment initiation, the patient reported experiencing nausea and vomiting. Treatment continued while awaiting genetic testing results.

## Discussion

BFH is primarily composed of fibroblasts and histiocytes, exhibiting benign yet diverse histological features (2, 6). Common variants include cellular, aneurysmal, and atypical forms, along with less frequent variants such as lipidized, palisaded, and myxoid types (6). These subtypes differ in pathology and prognosis. For example, cellular FH is characterized by larger cells, higher density, and less intercellular collagen, whereas atypical FH displays a mixture of pleomorphic spindle and polygonal cells with multinucleated giant cells, prominent nucleoli, and hemosiderin deposits (10, 11). Immunohistochemistry aids in the differentiation and diagnosis of BFH from other benign lesions, such as neurofibromas, leiomyomas, amelanotic melanomas, and dermatofibrosarcoma protuberans (DFSP). DFSP typically exhibits high CD34 expression, while BFH is often CD34-negative (2, 6, 12–14). Smooth muscle actin (SMA) is a marker for smooth muscle tumors and is generally negative in BFH but positive in leiomyomas (15). Amelanotic melanomas often show diffuse S-100 positivity, whereas BFH is S100 negative (2). In our cases, immunohistochemical analyses of both primary and metastatic lesions were performed to confirm tumor type and metastasis. Diagnostic markers used included Vimentin, S100, SMA, CD34, CD68, CD163, Desmin, AE1/AE3, and Ki67. AE1/AE3 serves as an epithelial tumor marker, Vimentin identifies fibroblasts, CD68 highlights monocytes, histiocytes, osteoclasts, mast cells, and giant cells, and Desmin is specific for skeletal muscle tumors. Beyond morphology and immunophenotype, recent studies point to several molecular clues related to metastasis. In general, metastasizing BFH carries a higher burden of copy-number alterations, most often gains of chromosome 7 and 8q and loss of Xq (10). PKC-related gene fusions such as CD63::PRKCD have also been documented, particularly in aneurysmal lesions, and metastasizing specimens may display recurrent gains of chromosomes 1, 3, 8, and 11 (16).

BFH predominantly occurs in the extremities of middle-aged adults, although it can develop in any anatomical site and at any age. Different subtypes share similar predilection sites and age ranges (2, 11, 17). Recurrence risks vary by subtype. Conventional FH and most variants rarely recur even with incomplete excision (1-2%). However, cellular, aneurysmal, and atypical BFH have recurrence rates as high as 20% (10, 11, 17, 18). Metastasis of BFH is exceedingly rare, and most cases reported involve the lungs and lymph nodes. Although histological features associated with recurrence and metastasis include larger size, aneurysmal changes, high cellularity, pleomorphism, high mitotic rates, and necrosis (6), subsequent studies suggest that morphology alone cannot reliably predict aggressive behavior (5, 10). Studies indicate that recurrent or large-cell variants of FH are more prone to metastasis (5). Among the three patients detailed in this study, all exhibited BFH metastasis and multiple recurrences. One case involved a rare occipital primary BFH with submental and pulmonary metastases, later progressing to widespread soft tissue metastases. The other two cases demonstrated lymph node metastases, with one patient also exhibiting subcutaneous metastasis. These patterns of metastasis are clinically uncommon.

Surgical excision remains the primary treatment for BFH. Most patients achieve a cure through surgery. However, when tumor location or disease burden makes resection infeasible or unacceptably morbid, radiotherapy or systemic therapy should be considered in consultation with a multidisciplinary team. In the absence of formal guidelines for radiotherapy or chemotherapy in BFH, management is typically extrapolated from other soft-tissue tumors. To date, these modalities have not shown clear efficacy. A case report described a patient with aneurysmal-type FH and metastasis who could not undergo surgery due to tumor vascularity and disease severity (19). This patient received palliative radiotherapy and chemotherapy without success, ultimately succumbing to the disease. Another patient with pulmonary metastases received chemotherapy, starting with ifosfamide and then single-agent doxorubicin (20). The disease progressed despite both treatments. One case series reported five patients who received chemotherapy and five who received radiotherapy (5). Chemotherapy regimens included doxorubicin–ifosfamide, sorafenib, and gemcitabine. Four of the five patients experienced disease progression. Among those treated with radiotherapy, one patient died. Thus, disease-specific radiotherapy protocols and effective systemic therapies for BFH remain undefined, highlighting the need for therapeutic development and prospective evaluation.

Compared with prior publications—most of which are single-case reports or very small series (2, 4, 6, 8, 21)—our study offers several advantages. First, we report a single-center cohort of 10 patients collected over 15 years with uniform pathology review, enhancing internal comparability. Second, all cases are previously unreported. Third, we expand the clinicopathologic spectrum by documenting uncommon presentations and atypical metastatic patterns, notably multifocal subcutaneous deposits. Fourth, we integrate radiologic and pathologic assessment and apply a pragmatic immunohistochemical framework to both primary and metastatic lesions, helping to reduce misclassification between BFH and other soft-tissue tumors. Fifth, we report real-world experience with multimodal combined therapy, including various permutations of surgery, radiotherapy, and chemotherapy. Using patient-level timelines, we delineate recurrence windows and initial metastatic sites. These observations inform treatment strategies for patients with metastatic BFH. Such integrated information is rarely consolidated in prior reports. Finally, we propose core data elements for standardized reporting to facilitate cross-study comparisons and future registry-based aggregation. Taken together, these features make our series a more informative, practice-oriented reference than the existing literature on metastatic BFH.

## Data Availability

The original contributions presented in the study are included in the article/Supplementary Material. Further inquiries can be directed to the corresponding author.
