# Supplementary material for: Case Report: Metastatic benign fibrous histiocytoma: a case series and review of diagnostic and therapeutic challenge
Source: Front Oncol. 2025 Nov 17;15:1621760. doi: 10.3389/fonc.2025.1621760 (PMC12665544; doi:10.3389/fonc.2025.1621760)
Supplement: Supplementary file 1 [file Presentation1.pptx]

## Slide 1
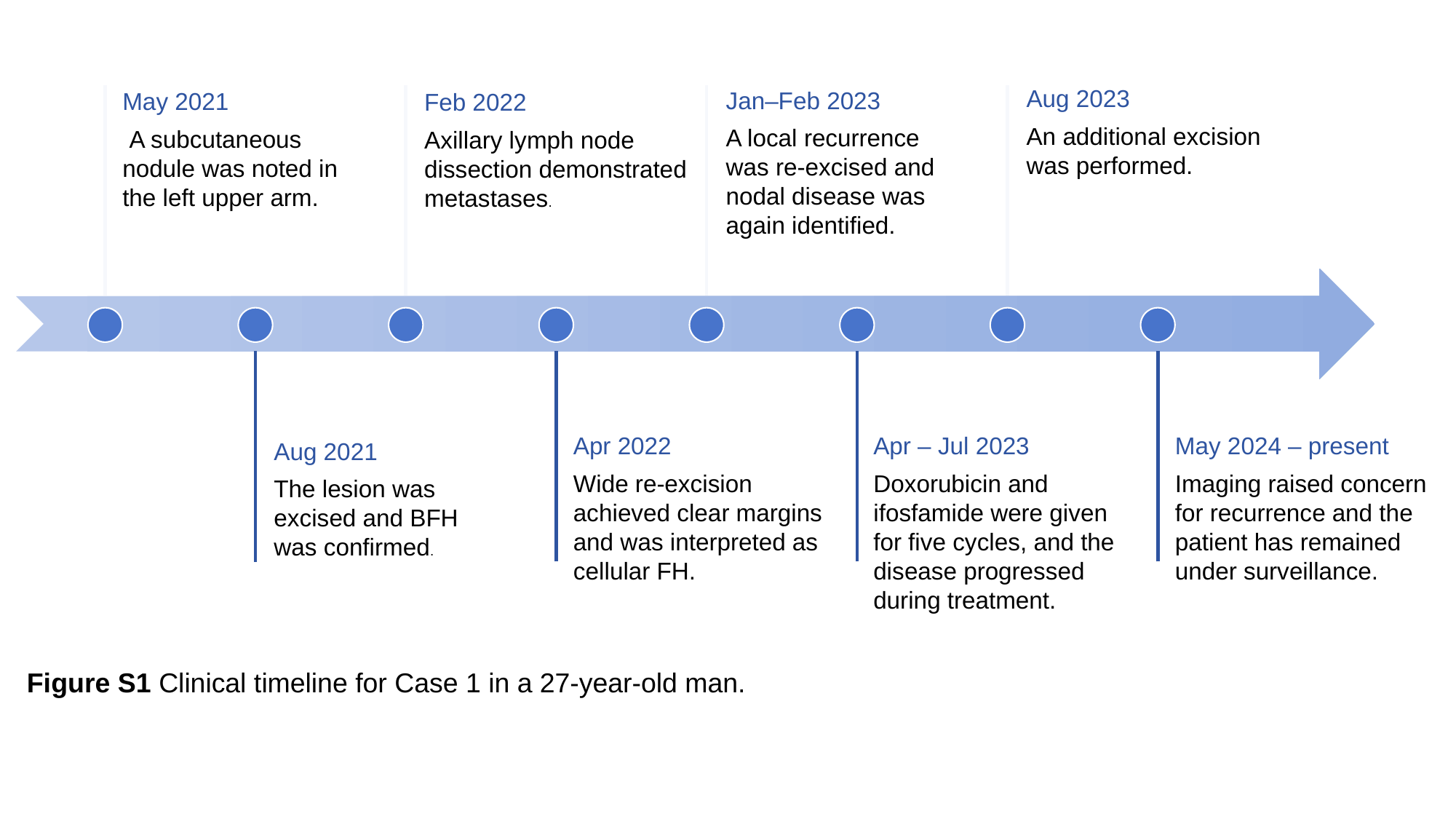

May 2021
 A subcutaneous nodule was noted in the left upper arm.
Jan–Feb 2023
A local recurrence was re-excised and nodal disease was again identified.
Aug 2023
An additional excision was performed.
Feb 2022
Axillary lymph node dissection demonstrated metastases.
Apr 2022
Wide re-excision achieved clear margins and was interpreted as cellular FH.
Apr – Jul 2023
Doxorubicin and ifosfamide were given for five cycles, and the disease progressed during treatment.
May 2024 – present
Imaging raised concern for recurrence and the patient has remained under surveillance.
Aug 2021
The lesion was excised and BFH was confirmed.
Figure S1 Clinical timeline for Case 1 in a 27-year-old man.
