# Supplementary material for: Case Report: Metastatic benign fibrous histiocytoma: a case series and review of diagnostic and therapeutic challenge
Source: Front Oncol. 2025 Nov 17;15:1621760. doi: 10.3389/fonc.2025.1621760 (PMC12665544; doi:10.3389/fonc.2025.1621760)
Supplement: Supplementary file 2 [file Presentation2.pptx]

## Slide 1
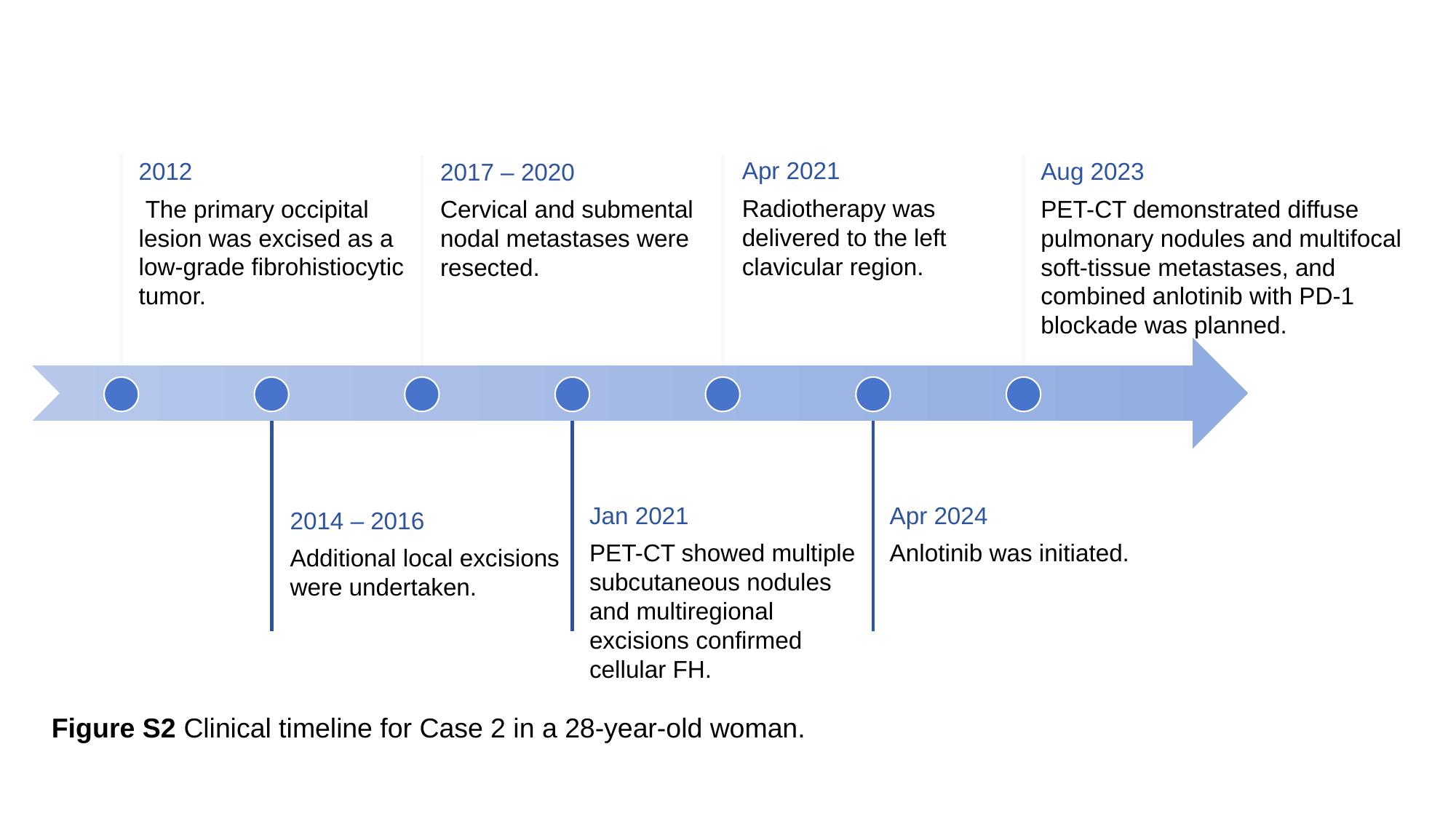

Aug 2023
PET-CT demonstrated diffuse pulmonary nodules and multifocal soft-tissue metastases, and combined anlotinib with PD-1 blockade was planned.
Apr 2021
Radiotherapy was delivered to the left clavicular region.
2017 – 2020
Cervical and submental nodal metastases were resected.
2012
 The primary occipital lesion was excised as a low-grade fibrohistiocytic tumor.
Jan 2021
PET-CT showed multiple subcutaneous nodules and multiregional excisions confirmed cellular FH.
Apr 2024
Anlotinib was initiated.
2014 – 2016
Additional local excisions were undertaken.
Figure S2 Clinical timeline for Case 2 in a 28-year-old woman.
