# Supplementary material for: Case Report: Metastatic benign fibrous histiocytoma: a case series and review of diagnostic and therapeutic challenge
Source: Front Oncol. 2025 Nov 17;15:1621760. doi: 10.3389/fonc.2025.1621760 (PMC12665544; doi:10.3389/fonc.2025.1621760)
Supplement: Supplementary file 3 [file Presentation3.pptx]

## Slide 1
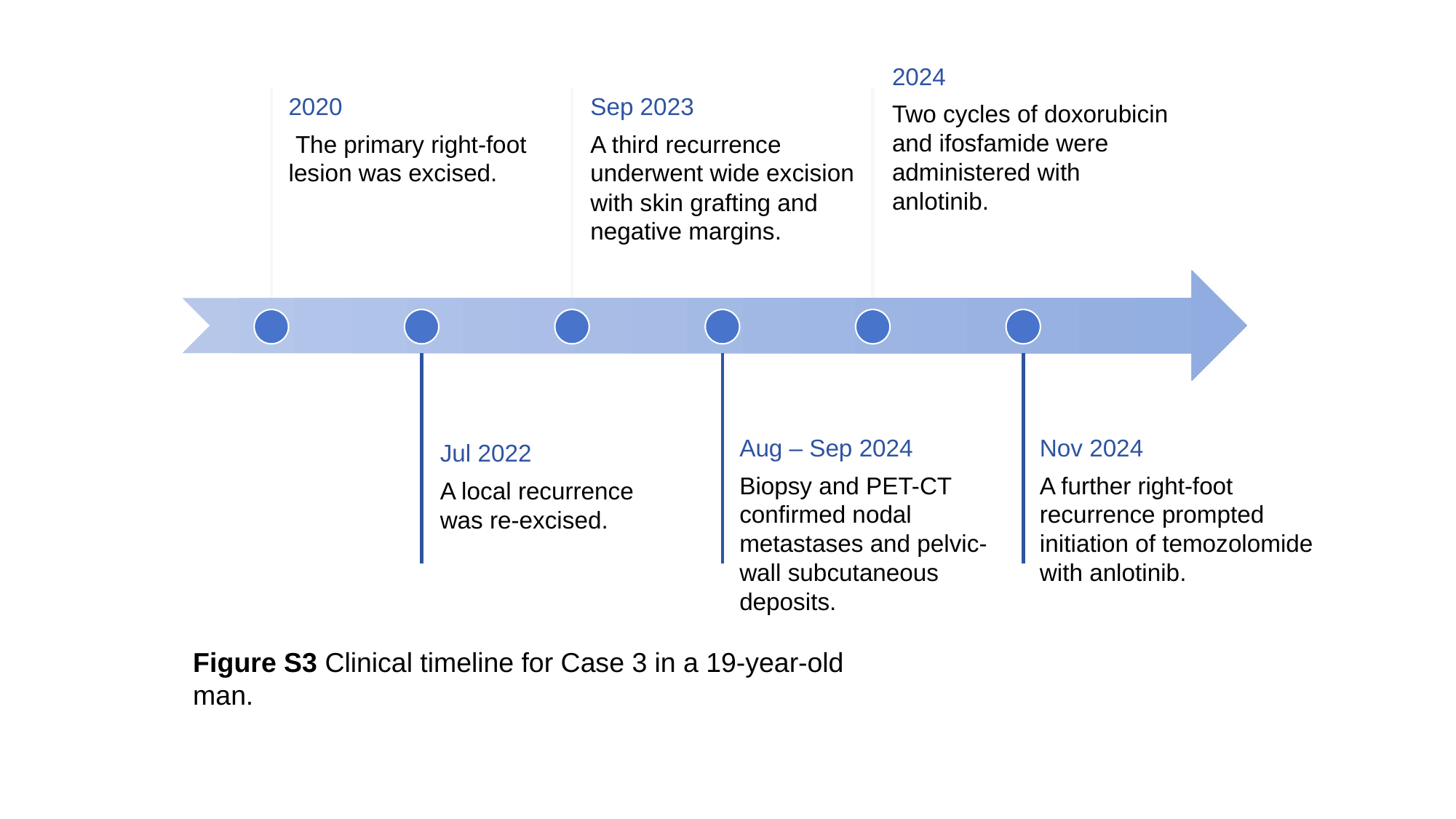

2024
Two cycles of doxorubicin and ifosfamide were administered with anlotinib.
Sep 2023
A third recurrence underwent wide excision with skin grafting and negative margins.
2020
 The primary right-foot lesion was excised.
Aug – Sep 2024
Biopsy and PET-CT confirmed nodal metastases and pelvic-wall subcutaneous deposits.
Nov 2024
A further right-foot recurrence prompted initiation of temozolomide with anlotinib.
Jul 2022
A local recurrence was re-excised.
Figure S3 Clinical timeline for Case 3 in a 19-year-old man.
